# Dysregulation of lncRNAs in Rheumatoid Arthritis: Biomarkers, Pathogenesis and Potential Therapeutic Targets

**DOI:** 10.3389/fphar.2021.652751

**Published:** 2021-03-12

**Authors:** Chenggui Miao, Liangliang Bai, Yaru Yang, Jinling Huang

**Affiliations:** ^1^ Department of Pharmacology, School of Integrated Chinese and Western Medicine, Anhui University of Chinese Medicine, Hefei, China; ^2^ Anhui Provincial Key Laboratory of Chinese Medicine Compound, Anhui University of Chinese Medicine, Hefei, China; ^3^ Department of Pharmacy, School of Life and Health Sciences, Anhui University of Science and Technology, Fengyang, China; ^4^ Department of Biomedical Engineering, School of Biomedical Engineering, Anhui Medical University, Hefei, China; ^5^ Department of Pharmacy, First Affiliated Hospital, Anhui Medical University, Hefei, China

**Keywords:** rheumatoid arthritis, long noncoding RNAs, fibroblast-like synoviocytes, inflammation, epigenetic modification

## Abstract

Rheumatoid arthritis (RA) is a chronic autoimmune disease of unknown etiology, mainly manifested by persistent abnormal proliferation of fibroblast-like synoviocytes (FLSs), inflammation, synovial hyperplasia and cartilage erosion, accompanied by joint swelling and joint destruction. Abnormal expression or function of long noncoding RNAs (lncRNAs) are closely related to human diseases, including cancers, mental diseases, autoimmune diseases and others. The abnormal sequence and spatial structure of lncRNAs, the disorder expression and the abnormal interaction with the binding protein will lead to the change of gene expression in the way of epigenetic modification. Increasing evidence demonstrated that lncRNAs were involved in the activation of FLSs, which played a key role in the pathogenesis of RA. In this review, the research progress of lncRNAs in the pathogenesis of RA was systematically summarized, including the role of lncRNAs in the diagnosis of RA, the regulatory mechanism of lncRNAs in the pathogenesis of RA, and the intervention role of lncRNAs in the treatment of RA. Furthermore, the activated signal pathways, the role of DNA methylation and other mechanism have also been overview in this review.

## Introduction

Rheumatoid arthritis is a chronic autoimmune disease of unknown etiology (Madav et al., 2020). RA is characterized by abnormal synovial hyperplasia, cartilage erosion and chronic joint inflammation of the hand and foot joints, often accompanied by other organ diseases and positive serum rheumatoid factor, which eventually leads to joint deformities and loss of function (Weyand and Goronzy, 2020).

The pathogenesis of RA is still unclear, but it is most likely related to the unique anatomy and physiological structure of the joint (Alpizar-Rodriguez and Finckh, 2020). The proliferation of fibroblast-like synoviocytes, the invasion of lymphocytes, and the formation of microvessels cause the synovial membrane to invade the cartilage surface to form pannus, destroying the structure and function of bone and cartilage (Nygaard and Firestein, 2020). Activated FLSs induce the chronic inflammation of synovium and bone erosion, and play an important role in the pathogenesis of RA (Pap et al., 2020).

Long non-coding RNAs (lncRNAs) are non-coding RNAs with length greater than 200 nucleotides (Weidle et al., 2017). lncRNAs play an important role in many life activities such as epigenetic regulation, cell cycle regulation and cell differentiation, and have become a research hotspot in genetics (Wei et al., 2017). As important regulators of pathological and physiological process, lncRNAs are also central regulators of inflammatory response, but they are poorly conserved among species (Mathy and Chen 2017).

LncRNAs can positively or negatively regulate the corresponding coding genes through various molecular mechanism (Hombach and Kretz, 2016). For example, lncRNAs can induce miRNA sponges, recruit proteins that directly enhance or interfere with transcription, and recruit chromatin modifiers, such as the polycomb repressor complexes (PRC), histone demethylases and DNA methyltransferases (Sarkar et al., 2015).

The involvement of lncRNAs in the pathogenesis of RA has been confirmed by more and more evidence (Dolcino et al., 2019). For example, Luo et al. (2017) found that there were 5,045 disordered lncRNAs in peripheral blood mononuclear cells (PBMCs) of RA patients (2,410 up-regulated and 2,635 down-regulated) compared with control. Among these lncRNAs, there were 135 potential lncRNA-mRNA target pairs, and RP11-498C9.15 targeted RA-related signaling pathways and genes closely related to RA pathogenesis in the genome.

In view of the important role of lncRNAs in RA, this work systematically summarized the new research progress of lncRNAs in RA pathogenesis, including the roles of lncRNAs in RA diagnosis, the regulatory mechanism of lncRNAs in RA pathogenesis, and the intervention effect of lncRNAs in RA treatment (Table 1).

**TABLE 1:** Aberrant lncRNAs reported in RA pathogenesis.

miRNA	Change	Tissue or cell type	Regulatory role	Targets	References
IFNG-AS1	Up-regulation	Peripheral blood and CD4^+^ T cells of RA patients	Increased IFNG-AS1 plays an important role in RA by regulating the IFNG.	IFNG	Peng et al. (2020)
Lnc-IL7R	Up-regulation	FLSs of RA patients	Lnc-IL7R promotes the growth of FLSs through interaction with EZH2	EZH2	Ye et al. (2017)
LINC00152	Up-regulation	FLSs of RA patients	FOXM1 activates the LINC00152 expression and induces the activation of the Wnt signaling	miR-1270	Wang et al., 2020e)
GAPLINC	Up-regulation	FLSs of RA patients	GAPLINC promotes tumor-like biologic behaviors of FLSs as miRNA sponging in RA patients	miR-382-5p and miR-575	Mo et al. (2018)
DILC	Down-regulation	Plasma and FLSs of RA patients	DILC participates in RA by inducing apoptosis of FLSs and down-regulating IL-6	IL-6	Wang et al. (2020a)
UCA1	Down-regulation	FLSs of RA patients	UCA1 affects the survival ability of FLSs by changing the expression of Wnt6	Wnt6	Yan et al. (2018)
GAS5	Down-regulation	Plasma and FLSs of RA patients	GAS5 overexpression down-regulates IL-18 and induces the apoptosis of FLSs	IL-18	Ma et al. (2019)
ZFAS1	Up-regulation	FLSs of RA patients	ZFAS1 promoted FLS migration and invasion in a miR-27a-dependent manner	miR-27a	Ye et al. (2018)
RP11–83J16.1	Up-regulation	Synovial tissue, synovial fluid and FLSs of RA patients	RP11–83J16.1 promotes FLS proliferation, migration, invasion and inflammation by regulating URI1	URI1	Piao et al. (2020)
PICSAR	Up-regulation	FLSs and synovial fluid from RA patients	PICSAR promotes cell proliferation, migration and invasion of FLSs by sponging miRNA-4701-5p	miRNA-4701-5p	Bi et al. (2019)
THRIL	Up-regulation	Serum, synovial tissue and FLSs of RA patients	THRIL regulates FLS growth and inflammatory response by activating the PI3K/AKT signaling	PI3K/AKT signaling pathway	Liang et al. (2020)
LINC01197	Down-regulation	Synovial tissue and FLS of RA model mice	LINC01197 sponges miR-150 to promote THBS2 expression and TLR4/NF-κB inactivation	miR-150	Zhao et al. (2020)
C5T1lncRNA	Up-regulation	Various tissue and PBMCs of RA patients	C5T1lncRNA is located in the associated region and influences transcript levels of C5	C5 mRNA	Messemaker et al. (2016)
*NTT*	Up-regulation	PBMCs of the first diagnosed untreated early RA patients	The excessive activation of the lncRNA NTT/PBOV1 axis promoted the monocyte differentiation of RA	PBOV1	Yang et al. (2018)
MEG3	Down-regulation	FLSs and chondrocytes from RA patients	MEG3 inhibits RA through miR-141 and AKT/mTOR signaling pathway	miR-141, AKT/mTOR signaling	Li et al. (2019a)
MEG3	Down-regulation	Serum of RA patients	MEG3 gene rs941576 (A/G) polymorphism was associated with increased severity of RA.	HIF-1α and VEGF	Wahba et al. (2020)
HOTAIR	Down-regulation	LPS-treated chondrocytes and RA mice	HOTAIR alleviates the pathological development of RA by targeting miR-138 and NF-κB pathway	miR-138 and NF-κB pathway	Zhang et al. (2017a)
LINC01882	Down-regulation	T Cells of RA patients, Jurkat T cells	LINC01882 is related to T cell activation and played an important role in RA.	IL-2	Houtman et al. (2018)
NEAT1	Up-regulation	PBMCS and Th17 cells from RA patients	NEAT1 promoted the differentiation of CD4^+^ T cells into Th17 cells	STAT3	Shui et al. (2019)
HIX003209	Up-regulation	PBMCs and macrophages of RA	HIX003209 promotes RA inflammation by sponging miR-6089 via TLR4/NF-κB signaling pathway	miR-6089	Yan et al. (2019)
H19	Up-regulation	Synovial tissue and FLS from patients with RA and from mice	Activated DDR-2 induces the expression of H19 and H19 directly interacts with and promotes the degradation of miR-103a	miR-103a	Mu et al. (2020)
LERFS	Down-regulation	FLSs and synovial tissue from RA patients and model rats	LERFS negatively regulates the migration, invasion, and proliferation of FLS.	hnRNP Q	Zou et al. (2018)
FER1L4	Down-regulation	FLSs and synovial tissue from RA patients	FER1L4 regulates RA via targeting NLRC5 potentially	NLRC5	Yu et al. (2020)
GAS5	Down-regulation	Synovial tissue and FLSs of RA patients	Overexpression of GAS5 reduced the levels of HIPK2, TNF-α and IL-6 by targeting the HIPK2	HIPK2	Li et al. (2020b)
MALAT1	Down-regulation	Synovial tissue and FLSs of RA patients	MALAT1-driven inhibition of Wnt signal impedes proliferation and inflammation	CTNNB1 promoter	Li et al. (2019b)
HOTTIP	Up-regulation	FLSs of RA patients	HOTTIP promotes inflammation in RA by methylation of SFRP1	SFRP1	Hu et al. (2020b)
PVT1	Up-regulation	FLSs and synovial tissue of RA model rats	PVT1 knockdown suppresses FLS inflammation and induces apoptosis in RA.	sirt6	Zhang et al. (2019a)
lncRNA-p21	Down-regulation	Blood samples of RA patients, primary and transformed cell lines	Methotrexate induces the lncRNA-p21, reduced NF-κ B activity in TNFα treated cells	NF-κ B	Spurlock et al. (2014)
GAS5	Down-regulation	Synovial tissue and FLS of RA patients	Tanshinone IIA could increase the expression of GAS5, promote the apoptosis of RA FLS	PI3K/Akt signaling pathway	Li et al. (2018a)
MALAT1	Down-regulation	FLSs of RA patients	MALAT1 induces apoptosis by inhibiting the activation of the PI3K/AKT pathway	PI3K/AKT pathway	Pan et al. (2016)
uc.477	Up-regulation	Serum and FLSs of RA patients, RA model mice	HQT on RA are closely related to its modulation of lncRNA uc.477	miR-19b	Wang et al. (2020d)
MEG3	Down-regulation	FLSs and synovial tissue from CFA-induced RA model rats	MEG3 regulates rheumatoid arthritis by targeting NLRC5	NLRC5	Liu et al. (2019)

## LncRNAs as Circulating Rheumatoid Arthritis Diagnostic Markers

Compared with healthy control, the expression of lncRNA-Cox2 in the serum of RA patients were significantly up-regulated, and the levels of IL-6 and MMP-9 were also significantly higher than those of healthy subjects (Shaker et al., 2019). lncRNA-Cox2 and HOTAIR can be used as new serum biomarkers to distinguish RA patients from healthy individuals (Bian et al., 2019; Lao and Xu, 2020).

The transcription level of lncRNA IFNG-AS1 in the peripheral blood of RA patients was increased, and the increased IFN-AS1 transcription level was strongly positively correlated with the levels of rheumatoid factor, erythrocyte sedimentation rate (ESR) and C-reactive protein (CRP) (Fang et al., 2020). IFNG as a target gene of IFNG-AS1 was overexpressed in RA patients, and it was positively correlated with the transcription level of IFNG-AS1. Furthermore, T-bet regulated the transcription of IFNG-AS1 in human CD4^+^ T cells. The up-regulation of T-bet transcription level was also positively correlated with the expression of IFNG-AS1. Under the guidance of T-bet, the increased IFNG-AS1 played an important role in the pathogenesis of RA by regulating the expression of IFNG (Padua et al., 2016; Peng et al., 2020).

Qin et al. (2019) used Agilent LncRNA + mRNA human gene expression microarray V4.0 to characterize the plasma lncRNA expression profile of RA patients. The co-expression network constructed included 229 network nodes and 340 connections between 116 lncRNAs and 113 mRNAs. Compared with control, the levels of 289 lncRNAs in the plasma of RA patients changed significantly, of which 169 were up-regulated and 120 were down-regulated. This further suggests that lncRNAs are involved in the pathogenesis of RA.

**FIGURE 1:**
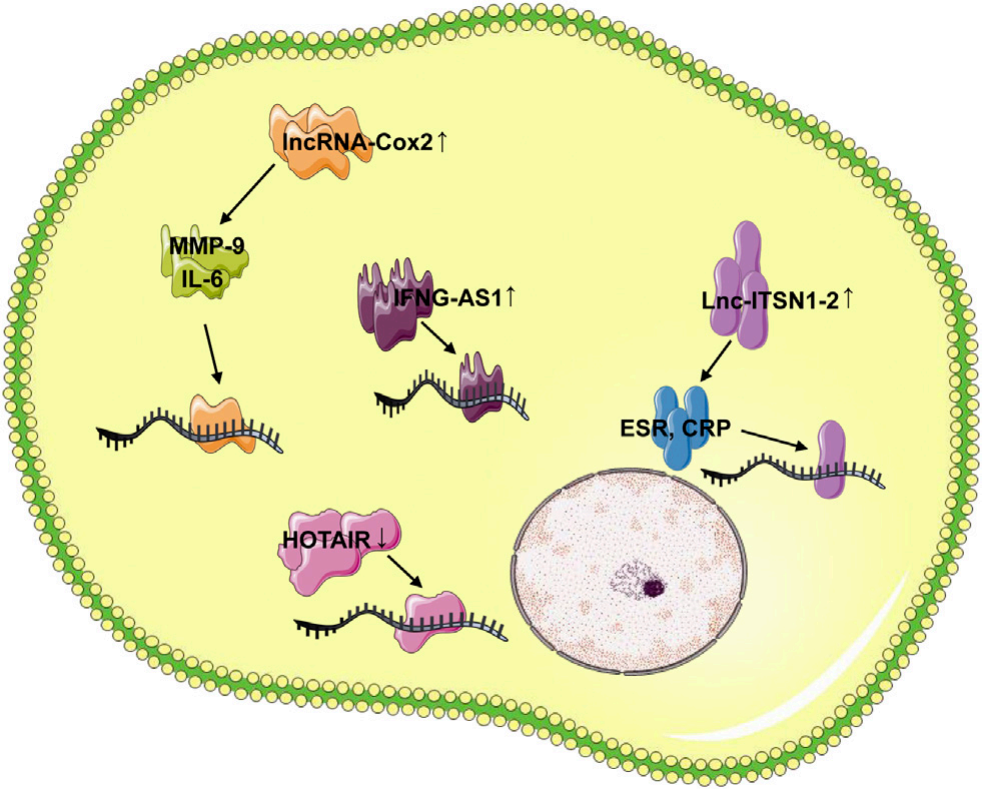
LncRNAs as circulating RA diagnostic markers. In serum of RA patients, the lncRNA-Cox2, lncRNA IFNG-AS1 and lnc-ITSN1-2 were up-regulated, whereas the HOTAIR expression was down-regulated. The lncRNA-Cox2 was involved in the pathogenesis of RA by targeting IL-6 and MMP-9, the increased IFNG-AS1 played important role in RA pathogenesis by regulating the IFNG, and the lnc-ITSN1-2 was positively correlated with ESR, CRP and disease activity score.

In addition, the level of lnc-ITSN1-2 in the plasma of RA patients was significantly increased compared with control. Plasma lnc-ITSN1-2 levels were positively correlated with ESR, CRP and disease activity score (Gong et al., 2017). Circulating lnc-ITSN1-2 has a high diagnostic value for RA, and the disordered expression of lnc-ITSN1-2 may be a new marker for RA diagnosis and disease treatment (Lee and Bae, 2018; Yue et al., 2019) (Figure 1).

## LncRNAs Involved in the Pathogenesis of Rheumatoid Arthritis

As the research spread, a large number of lncRNAs were found to be disorderly expressed in RA synovial tissue and immune cells, mediating abnormal proliferation of FLSs, synovial inflammation, cartilage erosion, bone damage and abnormal immune response (Chen et al., 2019a; Waller and Blann, 2019; Xu et al., 2019).

Jiang et al. found as many as 260 differentially expressed lncRNAs in the synovium between the AA model and normal rats, with 170 up-regulated and 90 down-regulated. Six LncRNAs, XR_008357, U75927, MRAK046251, XR_006457, DQ266363 and MRAK003448 might play crucial role in the pathogenesis of RA (Jiang et al., 2016).

Zhang et al. (2016) found that 135 lncRNAs were disordered in FLSs between RA patients and healthy individuals. The level of lncRNA ENST00000483588 increased significantly, while the levels of three lncRNAs (ENST00000438399, uc004afb.1 and ENST00000452247) decreased significantly. Among them, the level of ENST00000483588 was positively correlated with the level of CRP. Jiang et al. (2017) found that three lncRNAs (S5645.1, XR_006437.1, J01878) were highly correlated with RA, and these three lncRNAs might be potential diagnostic biomarkers and therapeutic targets.

In addition, lnc-AL928768.3 and lnc-AC091493.1 increased in RA patients compared with the control group, and these two lncRNAs were positively correlated with ESR, CRP levels and disease activity scores (Sun et al., 2020b). lnc-AL928768.3 and lnc-AC091493.1 may be new markers of RA risk and disease severity.

The detection of lncRNAs levels on the synovial tissue sample of patients with RA collected during surgery can best reflect the change of lncRNAs with the pathological development, and help to investigate the regulatory mechanism of lncRNAs in the pathogenesis of RA.

### LncRNAs Involved in Fibroblast-Like Synoviocytes Regulation

FLS activation and proliferation play key role in the pathogenesis of RA. Abnormal proliferation of FLS release IL-6, IL-8, IL-15 and other cytokines and chemokines, and promote the migration and activation of leukocytes from blood vessel to synovium (Miao et al., 2014; Miao et al., 2015b). FLS synthesize and secrete extracellular matrix protein such as fibronectin and cell adhesion molecule, and recruit and reside leukocytes in synovial tissue. FLS can activate B lymphocytes, secrete matrix metalloproteinase-3 and matrix degrading enzyme, degrade articular cartilage and aggravate RA (Miao et al., 2018b; Miao et al., 2018c).

#### Fibroblast-Like Synoviocytes Proliferation

Long noncoding-interleukin-7 receptor (lnc-IL7R) interacted with the enhancer of zeste homolog 2 (EZH2) to promote FLS proliferation and cell cycle progression in RA. Furthermore, lnc-IL7 was necessary for PRC2-mediated inhibition of the cyclin-dependent kinase inhibitor 1A and 2A (Ye et al., 2017).

The level of cytoskeleton regulator RNA (LINC00152) was significantly increased in RA FLSs compared with control. The enhanced LINC00152 promoted the proliferation of RA FLSs by inducing the activation of the canonical Wnt signaling pathway. Furthermore, forkhead box M1 (FOXM1) was the upstream regulator of LINC00152 that transcriptionally activated the LINC00152 expression, leading to the activation of the Wnt signaling. Surprisingly, LINC00152 positively regulated the FOXM1 by making miR-1270 spongy. The function of FOXM1/LINC00152 feedback loop in the regulation of RA FLSs was instructive for us to investigate the complex pathogenesis of RA (Fattahi et al., 2020; Wang et al., 2020e).

In addition, the newly identified functional LncRNA in oncology GAPLINC promoted the FLS tumor-like behavior in miR-382-5p and miR-575-dependent manner in FLSs of RA patients. GAPLINC silencing increased the expression of miR-382-5p and miR-575, while GAPLINC overexpression had the opposite effect. GAPLINC may be a new and valuable therapeutic target for RA patients (Liao et al., 2018; Mo et al., 2018).

#### Fibroblast-Like Synoviocytes Apoptosis

LncRNA DILC regulated liver cancer stem cells by inhibiting IL-6 (Wang et al., 2016; Huang et al., 2019). Compared with the healthy control, the plasma DILC of RA patients was down-regulated, while IL-6 was up-regulated, and the plasma DILC level was significantly negatively correlated with RA pathology. The overexpression of DILC promoted the inhibition of FLS apoptosis and IL-6 expression in RA patients, while DILC silencing has the opposite effect. DILC participated in the pathological mechanism of RA by inducing FLS apoptosis and down-regulating IL-6 (Wang et al., 2020a).

LncRNA UCA1 was highly expressed in FLSs of healthy control and decreased in FLSs of RA patients. The decreased expression of UCA1 increased the proliferation of FLSs, while overexpression of UCA1 inhibited the survival of FLSs. UCA1 affected the survival ability of FLSs by changing the expression of wnt6, suggesting that Wnt signaling pathway play important role in the pathology of RA (Yan et al., 2018).

Furthermore, the plasma growth arrest specific transcript 5 (GAS5) level of RA patients was significantly down-regulated compared with the healthy control, while the IL-18 level was significantly increased. In RA patients, there was a significant negative correlation between GAS5 and IL-18 level. Interestingly, this negative regulatory relationship was not found in control. The overexpression of GAS5 in RA FLSs resulted in the inhibition of IL-18 expression, leading to the promotion of FLS apoptosis (Ma et al., 2019). This regulation through GAS5 overexpression may help the treatment of RA disease.

In addition to low-expressed lncRNAs, high-expressed lncRNAs related to FLS apoptosis were also found during RA. LncRNA plasmacytoma variant translocation 1 (PVT1) in the synovial tissue of RA patients and RA model rats was significantly increased. PVT1 specifically bound to miR-543 and positively regulated the expression of signal peptide-CUB-EGF-like containing protein 2 (SCUBE2) by inhibiting the miR-543, leading to IL-1β secretion and FLS apoptosis inhibition. PVT1 inhibition may be a new idea for the treatment of RA (Wang et al., 2020b).

#### Fibroblast-Like Synoviocytes Migration and Invasion

LncRNA ZFAS1 has been observed to express significantly up-regulated in cancer, and the up-regulated ZFAS1 promoted the migration and invasion of cancer cells (Li et al., 2020c; Liu et al., 2020). Importantly, the expression of ZFAS1 in the synovial tissue and FLSs of RA patients also increased significantly. ZFAS1 knockout inhibited the migration and invasion of FLSs, while overexpression showed the effect of promoting the pathology of RA. ZFAS1 took miR-27a as a direct target and reduced the expression of miR-27a. Obviously, ZFAS1 promoted FLS migration and invasion in a miR-27a-dependent manner (Ye et al., 2018).

The expression of lncRNA RP11–83J16.1 in synovial tissue and FLSs of RA patients was significantly increased. The highly expressed RP11–83J16.1 used the URI1 as the target to regulate the expression of FRAT1 and β-catenin in FLSs, and induced the FLS proliferation, migration, invasion, inflammation (Piao et al., 2020).

LncRNA PICSAR is a lincRNA associated with skin squamous cell carcinoma (Piipponen et al., 2016). Compared with healthy control, the level of PICSAR in FLSs and synovial fluid of RA patients were significantly up-regulated. Increased PICSAR promoted the synovial invasion and joint destruction. After PICSAR expression was inhibited, FLS proliferation, migration, invasion and release of pro-inflammatory cytokines were also inhibited. PICSAR may play important role in sponging miR-4701-5p in RA and act as a marker of RA (Bi et al., 2019).

#### Synovial Inflammation

The highly expressed THRIL in the blood of RA patients was positively correlated with TNF-α level, DAS 28 and ESR. Inhibition of THRIL reversed the regulatory effect of TNF-α on RA FLSs, and significantly reduced the effect of TNF-α on the activity of phosphoinositide 3-kinase (PI3K) and *p*-AKT signaling pathways. Therefore, the highly expressed THRIL could promote the proliferation and inflammation of FLSs by activating the PI3K/AKT signaling pathway (Zhu et al., 2017; Liang et al., 2020).

**FIGURE 2:**
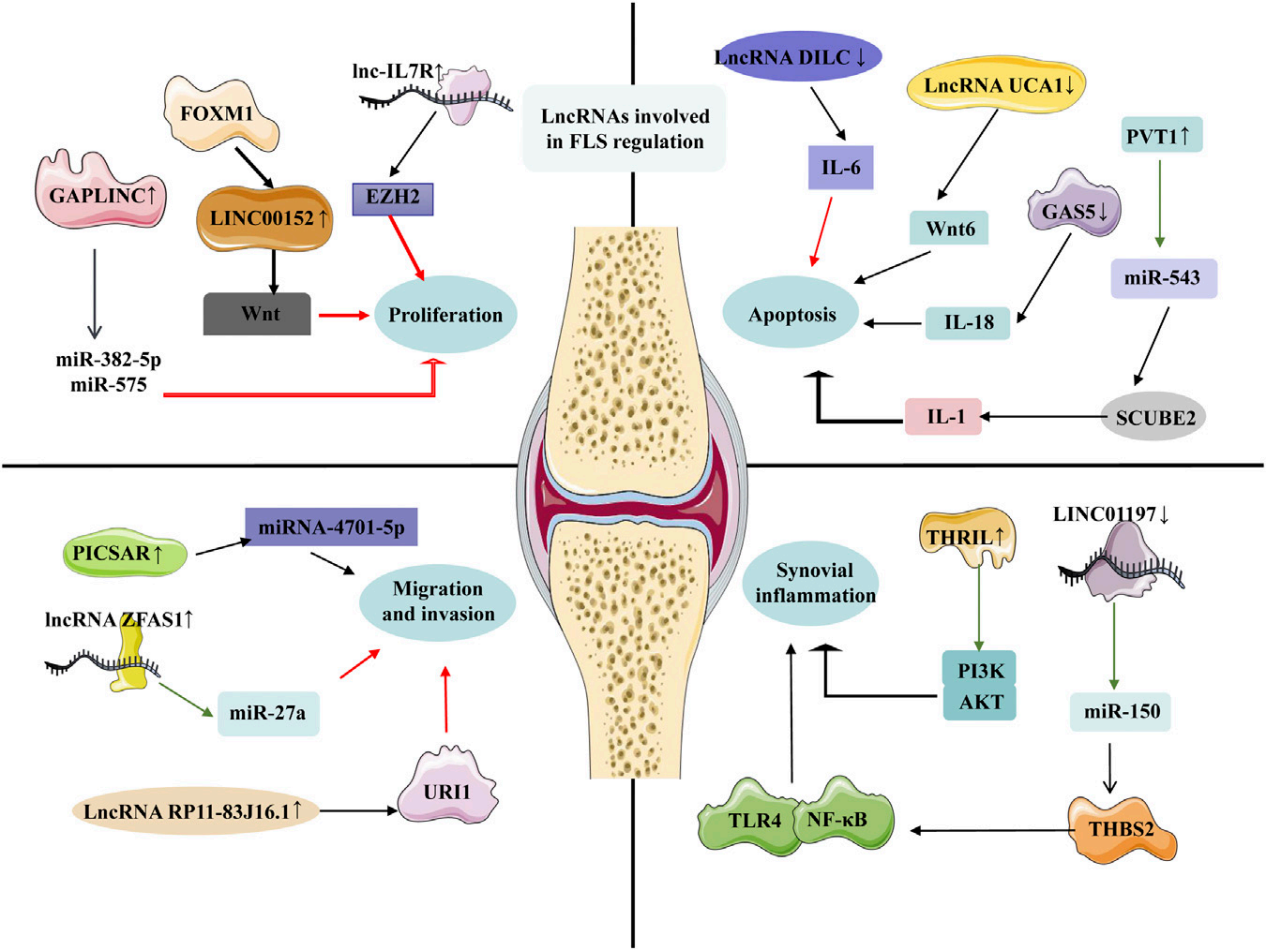
LncRNAs involved in FLS regulation. In RA FLS, the up-regulated lnc-IL7R interacted with the EZH2 to promote FLS proliferation and cell cycle progression, the FOXM1 was involved in the FLS proliferation by targeting the LINC00152, and the GAPLINC promoted the FLS tumor-like behavior in miR-382-5p and miR-575-dependent manners. The plasma DILC of RA patients was down-regulated, and the overexpression of DILC promoted the inhibition of FLS apoptosis by targeting the IL-6. LncRNA UCA1 was decreased, while overexpression of UCA1 inhibited the survival of FLSs. PVT1 specifically bound to miR-543 and positively regulated the expression of SCUBE2. The expression of LncRNA PICSAR, lncRNA ZFAS1, lncRNA RP11–83J16.1 in the synovial tissue and FLS of RA patients increased significantly, and these three lncRNAs affect the migration and invasion of FLS through their respective targets. The highly expressed THRIL in the blood of RA patients was positively correlated with TNF-α, DAS 28 and ESR, and the overexpression of LINC01197 inhibited the FLS proliferation, promoted the cell apoptosis, inhibited the synovial inflammation and reduced the severity of RA.

In addition, LINC01197 expression decreased in the synovial tissue of RA model mice compared with control. The overexpression of LINC01197 inhibited the FLS proliferation, promoted cell apoptosis, inhibited synovial inflammation, and reduced the severity of RA. MiR-150 was confirmed to be a direct target of LINC01197. LINC01197 promoted the expression of THBS2 by inhibiting the miR-150, which further led to the inactivation of the TLR4/NF-κB signaling pathway (Wang et al., 2017b; Zhao et al., 2020) (Figure 2).

### lncRNAs in PBMCS

Yuan et al. (2017) investigated the expression profile of lncRNAs in PBMCs of RA patients, and found that the ENST00000456270 and NR_002838 were up-regulated significantly, while the NR_026812 and uc001zwf.1 were down-regulated.

Wen et al. (Wen et al., 2020a; Wen et al., 2020b) found that in PBMCs of RA patients, the levels of seven lncRNAs (MIR22HG, DSCR9, LINC01189, MAPKAPK5-AS1, ENST00000619282, C5orf17 and LINC01006) were significantly changed compared with the control. Further analysis, MIR22HG, DSCR9, LINC01189, MAPKAPK5-AS1, and ENST00000619282 were potential biomarkers of RA, and their effect might be related to FLS apoptosis and autophagy. Messemaker et al. (Messemaker et al., 2015; Messemaker et al., 2016) determined the non-coding transcript (C5T1lncRNA) starting from the 3′’untranslated region (3′’UTR) of C5. The new lncRNA C5T1lncRNA was mainly expressed in the nucleus, and its expression was positively correlated with C5 mRNA in PBMCs, while C5T1lncRNA knockdown led to a decrease in C5 mRNA level, but did not affected the expression of other adjacent genes.

In addition, monocyte/macrophage differentiation mediates the inflammation and participates in the pathogenesis of RA (Paoletti et al., 2019). In the human monocytic leukemia cell line THP-1, the lncRNA noncoding transcript in T cells (NTT) was regulated by the key monocyte transcription factor C/EBPβ, and it bound to the promoter of the nearby gene PBOV1 through hnRNP-U. Overexpression of PBOV1 led to cell cycle G1 arrest and differentiation into macrophages. The C/EBPβ/NTT/PBOV1 axis was overactivated in PBMCs of the first diagnosed untreated early RA patients, which was consistent with the trend of higher disease activity DAS28 scores. The excessive activation of the lncRNA NTT/PBOV1 axis promoted the monocyte differentiation of RA and promoted the pathological development of this disease (Huber et al., 2012; Mousavi et al., 2018; Yang et al., 2018).

Single nucleotide polymorphism (SNP) plays key role in disordered lncRNA (Castellanos-Rubio and Ghosh, 2019). For example, Zhang et al. (2018) found that the level of lnc0640 in the PBMCs of RA patients was significantly increased compared with control, while the level of lnc5150 was significantly reduced. The disordered lnc0640 and lnc5150 were correlated with CRP, and the lnc5150 was correlated with the ESR. It was worth noting that the TT genotype of rs13039216 in the lnc0640 gene was related to the risk reduction of RA, and the G allele of the rs141561256 polymorphism in the lnc5150 gene was significantly related to the level of rheumatoid factor. It suggests that these two lncRNAs may be involved in the pathogenesis of RA, and SNPs play important role in the mechanism.

Identifying lncRNAs related to the transcription of RA risk genes in PBMCs has positive significance for RA.

### lncRNAs in Chondrocytes

The lncRNA maternally expressed gene 3 (MEG3) is a tumor suppressor involved in the pathogenesis of cancer (He et al., 2017). Compared with healthy control, the level of MEG3 in FLSs of RA patients was significantly down-regulated, and MEG3 was also significantly down-regulated in lipopolysaccharide (LPS)-treated chondrocytes (Wang et al., 2019a). In LPS-treated chondrocytes, the cell proliferation rate and the production of IL-23 were both inhibited, but this phenomenon was reversed in chondrocytes transfected with a lentivirus containing the MEG3 coding sequence. Importantly, It was worth noting that MEG3 was negatively correlated with miR-141 and AKT/mTOR signaling, and the effect of MEG3 overexpression was partially offset by overexpression of miR-141. The inhibitory effects of MEG3 overexpression on RA pathology might be achieved by the increase of chondrocyte proliferation rate, through the negative regulation of miR-141 and AKT/mTOR signaling pathway (Li et al., 2019a; Chen et al., 2019b; Lu and Qian, 2019).

SNP may be the cause of MEG3 disorder. The low expression of MEG3 in RA patients was negatively correlated with HIF-1α and vascular endothelial growth factor A (VEGF) serum level, and positively correlated with BAX. The polymorphism of MEG3 gene rs941576 (A/G) has been confirmed to be associated with the increased severity of RA in the current population (Wahba et al., 2020).

The level of LncRNA HOTAIR in the chondrocytes treated with LPS was significantly reduced. Overexpression of HOTAIR resulted in the up-regulation of the proliferation-related protein Ki67 and proliferating cell nuclear antigen (PCNA). There was a negative correlation between HOTAIR and miR-138 expression (Huang et al., 2017; Zhou et al., 2017). Overexpression of miR-138 partially reversed the effect of HOTAIR overexpression on proliferation and inflammation. Furthermore, HOTAIR overexpression inhibited the activation of NF-κB in LPS-treated chondrocytes by inhibiting p65 to the nucleus. The overexpression of HOTAIR also increased the cell proliferation of RA rats and inhibited inflammation through the reduction of CD4^+^ IL-17^+^, CD4^+^ IL-23^+^ cells and the down-regulation of IL-1β and TNF-α. HOTAIR alleviated the pathological development of RA by targeting miR-138 and NF-κB pathway, suggesting that HOTAIR might be a potential RA diagnostic marker and therapeutic target (Zhang et al., 2017a; Gupta et al., 2020).

Besides, HOTAIR directly inhibited the expression of WIF-1 by increasing the H3K27 trimethylation in WIF-1 promoter, leading to activation of the canonical Wnt pathway. Given that the pathway regulated the expression of MMP-13 and was responsible for the degradation of type II collagen in articular cartilage, HOTAIR was involved in the cartilage damage mechanism in RA pathology (Zhou et al., 2019). Obviously, HOTAIR plays important role in the regulation of cartilage cells in RA patients.

### lncRNAs in T Cells

LncRNA LINC01882 was mainly expressed in T cells, while the expression of LINC01882 was significantly down-regulated in anti-CD3/CD28 activated primitive CD4^+^ T cells. Knockdown of LINC01882 in Jurkat T cells showed that the expression of factors related to IL-2 regulation were up-regulated, such as the transcription factor ZEB1 and the kinase MAP2K4. LINC01882 was related to T cell activation and played important role in the abnormal immune mechanism of RA (Houtman et al., 2018).

lncRNA NEAT1 was significantly up-regulated in Th17 cells differentiated from CD4^+^ T cells *in vitro*. The up-regulation of NEAT1 promoted the differentiation of CD4^+^ T cells into Th17 cells by regulating its downstream molecule STAT3, whereas knockout of NEAT1 inhibited the differentiation and the pathological progress of RA by regulating the expression of STAT3. NEAT1 was an active molecule for CD4^+^ T cell differentiation, which was involved in the pathogenesis of RA (Mishra et al., 2019; Shui et al., 2019). The T cells of RA patients showed significantly elevated levels of GAS5, RMRP and THRIL compared with the control. A positive correlation was found between RMRP expression and disease duration in RA. GAS5, RMRP and THRIL have value in distinguishing RA patients from healthy individuals. (Wu et al., 2019a; Moharamoghli et al., 2019).

In addition to the five lncRNAs introduced above, seven up-regulated lncRNAs and two down-regulated lncRNAs were detected in peripheral blood CD4^+^ T cells from 12 active RA patients and eight healthy individuals using magnetic beads (Li et al., 2020a). These evidences showed that lncRNAs play important regulatory role in T cell abnormalities through corresponding mechanisms.

### lncRNAs Involved in Inflammation

Compared with the control, the level of LOC100652951 and LOC100506036 in the T cells of RA patients were significantly increased. The expression of LOC100652951 in T cells of RA patients treated with biological agents was significantly inhibited. Furthermore, the LOC100506036 was involved in regulating the expression of sphingomyelin phosphodiesterase 1 (SMPD1) and NFAT1 to promote the inflammatory response of RA (Lu et al., 2016).

The level of lncRNA HIX003209 was significantly up-regulated in the PBMCs of RA patients. Enhanced HIX003209 participated in TLR4-mediated inflammation by targeting miR-6089 in macrophages, and promoted the proliferation and activation of macrophages through the IκBα/NF-κB signaling pathway. HIX003209 exaggerated the inflammation of RA and promoted the pathological development of RA by sponging the miR-6089 through the TLR4/NF-κB pathway (Yan et al., 2019).

The level of H19 in the synovial tissue of RA patients was significantly higher than that of normal control. Both the MAP kinase ERK-1/2 pathway and the phosphatidylinositol 3-kinase pathway affected the H19 RNA expression. The increased sensitivity of overexpressed H19 RNA to starvation/cytokine regulation in RA indicated that embryo genes were involved in the pathogenesis of RA, which reflect the embryonic dedifferentiation and continuous inflammation in synovial tissue (Stuhlmüller et al., 2003; Jarroux et al., 2017; Yang et al., 2020).

Experimental analysis using mice showed that lncRNA GAS5 was maintained at a high basal level in immune organs (such as spleen and thymus), while its level in metabolic organs including liver, fat and skeletal muscle was lower. GAS5’s involvement in immune mechanism and inflammatory response may be achieved by inhibiting the mTOR pathway (Shi et al., 2013; Mayama et al., 2016).

### lncRNAs Involved in Joint Destruction

During the RA, the level of discoidin domain receptor 2 (DDR-2) was significantly increased, which was positively correlated with the level of interleukin and RA factor (Zhao et al., 2014). The activated DDR-2 induced the expression of H19 through c-Myc, and the enhanced H19 directly interacted with miR-103a and promoted its degradation. It could be confirmed that DDR-2 participated in the promotion of inflammation and joint destruction of RA through the regulation of H19-miR-103a axis and inflammatory factors (Mu et al., 2020; Wu et al., 2019b; Zhi et al., 2020).

Anticitrullinated protein antibody (ACPA)-negative RA is a subspecies of RA characterized by a milder disease (Shao et al., 2019). There were H3K4me3 histone markers, transcription factors and lncRNAs in rs2833522 located between HUNK and SCAF4. Rs2833522 was related to the severity of joint damage in ACPA-negative RA (de Rooy et al., 2015).

In addition, lncRNA LERFS negatively regulated the migration, invasion and proliferation of joint synovium by interacting with heterogeneous nuclear ribonucleoprotein Q (hnRNP Q). However, LERFS was low expressed in RA FLSs, and the reduced LERFS led to the reduction of LERFS-hnRNP Q complex, thereby reducing the binding of hnRNP Q to the mRNAs of small GTPase protein RhoA, Rac1 and CDC42 that control the activity and proliferation of FLSs. This mechanism increased the stability or translation of RA-related mRNAs, leading to synovial invasion and joint destruction in RA patients (Zou et al., 2018).

## LncRNAs That Interacts With DNA Methylation

DNA methylation is a form of DNA chemical modification, which can change the genetic performance without changing the DNA sequence. DNA methylation refers to the covalent binding of a methyl group to the cytosine 5 carbon site of CpG dinucleotide under the action of DNA methyltransferase. DNA methylation can cause changes in chromatin structure, DNA conformation, DNA stability and the interaction between DNA and protein, thus controlling gene expression (Miao et al., 2015a; Miao et al., 2018a). Studies have shown that DNA methylation is directly related to the regulation mechanism of lncRNAs.

### Low-Expressed lncRNAs

DNA methylation are involved in the roles of lncRNAs in the pathological mechanisms of RA, and the low level of lncRNAs may be related to the DNA methylation of its own promoter (Sun et al., 2020a).

LncRNA Fer-1-like protein 4 (FER1L4) was a reported tumor suppressor involved in cancers (Chen et al., 2018). Compared with healthy control, the level of FER1L4 in the synovial tissue and FLSs of RA patients was significantly reduced, and the NLRC5 was increased. Overexpression of FER1L4 inhibited the expression of NLRC5 and reduced the level of inflammatory cytokines. It is worth noting that the FER1L4 gene promoter was obviously methylated during the disease, and the methylation inhibitor 5-aza-2-deoxycytidine inhibited the FER1L4 promoter hypermethylation (Yu et al., 2020).

GAS5 was also involved in the proliferation and inflammatory response in RA. The expression of GAS5 in the synovial tissue and FLSs of RA patients was significantly reduced, while the expression of homeodomain-interacting protein kinase 2 (HIPK2) was significantly increased. The low expression of GAS5 related to hypermethylation in GAS5 promoter. Overexpression of GAS5 reduced the level of TNF-α and IL-6 by targeting the HIPK2 (Li et al., 2020b).

In normal physiological conditions, lung adenocarcinoma transcript 1 (MALAT1) bound to the CTNNB1 promoter region and recruited methyltransferase to promote the methylation of the CTNNB1 promoter (Li et al., 2018b). When CTNNB1 transcription was inhibited by methylation, the Wnt signaling pathway was blocked (Huangfu et al., 2018). In RA pathogenesis, silent MALAT1 could not methylate the CTNNB1 promoter, whereas stimulated the expression of β-catenin, increased the proliferation of FLSs and inhibited the apoptosis. MALAT1 was involved in the activation of Wnt pathway and the pathological progress of RA, and restored MALAT1 inhibited the secretion of inflammatory cytokines such as IL-6, IL-10 and TNF-α (Li et al., 2019b; Zhang et al., 2019b).

Previous studies have shown that nucleotide oligomerization domain (NOD)-like receptors 5 (NLRC5) plays key role in inflammation and autoimmune diseases (Zou and Xu, 2020). Our recent study showed that MEG3 level was significantly decreased, while NLRC5 was increased. The low expression of MEG3 was related to the abnormal increase of NLRC5 and inflammatory cytokines. Interestingly, methylation specific PCR showed that the promoter of MEG3 gene was significantly methylated. The methylation inhibitor 5-azadc could inhibit the hypermethylation of MEG3 promoter, which indirectly proved that DNA methylation was involved in the regulation of MEG3. These results suggest that epigenetic modification may regulate RA by targeting MEG3 and NLRC5 (Liu et al., 2019).

### Highly Expressed lncRNAs

In addition, highly expressed lncRNAs can bind to the promoter region of targets to recruit DNA methylases, trigger hypermethylation and inhibit their expression.

SFRP1 was upstream regulator of Wnt signaling pathway, which was decreased during RA (Miao et al., 2013; Miao et al., 2015c; Cici et al., 2019). LncRNA HOTTIP was highly expressed in FLSs of RA patients, silencing HOTTIP or SFRP1 overexpression inhibited the proliferation and invasion of RA FLS, and promoted the apoptosis. Evidence suggested that HOTTIP recruited Dnmt3b to the SFRP1 promoter, induced the hypermethylation of SFRP1 promoter and activated the Wnt signaling pathway (Hu et al., 2020b).

The PVT1 level was significantly increased in rat synovial tissue and FLSs, which was negatively regulated with the low expression of sirtuin 6 (sirt6). PVT1 in the nucleus bound to the sirt6 promoter and induced methylation of sirt6, leading to its transcriptional inhibition. PVT1 knockdown restored the expression of sirt6 by reducing the methylation of sirt6 promoter and reduced the level of RA factor (Zhang et al., 2019a).

## LncRNAs Involved in Drug Treatment of Rheumatoid Arthritis

### Methotrexate

Compared with healthy control, the level of long intergenic noncoding RNA-p21 (lncRNA-p21) in blood sample of RA patient was decreased, while the level of phosphorylated p65 (rela), a marker of NF-κ B activation, was significantly increased. Compared with RA patients who did not receive low-dose methotrexate treatment, the lncRNA-p21 in blood sample of the treatment group was increased, and the phosphorylated p65 (rela) was significantly decreased in the treatment group. In cell culture using primary and transformed cell lines, methotrexate induced lncRNA-p21 through a DNA dependent protein kinase catalytic subunit mechanism, which reduced NF-κ B activity in TNFα treated cells (Spurlock et al., 2014; Spurlock et al., 2015).

### Tocilizumab and Adalimumab

CD14^+^ monocytes were isolated from RA patients before and after treatment with anti-IL-6R (tocilizumab) or anti-TNF-α (adalimumab), and the transcriptional changes of lncRNAs were analyzed by microarray (Finzel et al., 2019). IL-6 or TNF-α treatment significantly regulated the expression of 85 lincRNAs, and the regulation of lincRNA transcription was highly specific to different cytokines. LincRNAs were involved in the mechanism of RA treatment with tocilizumab and adalimumab (Müller et al., 2014).

### Tanshinone IIA

LncRNA GAS5 in synovial tissue and FLSs of RA patients was significantly decreased. SiRNA knockout of GAS5 in FLSs from healthy control inhibited the apoptosis of RA FLSs and activated the PI3K/AKT signaling pathway. Tanshinone IIA could increase the expression of GAS5, promote the apoptosis of RA FLSs and inhibit the PI3K/Akt signaling pathway (Li et al., 2018a; Wang et al., 2019b; Tang et al., 2019). Tanshinone IIA may play a therapeutic role in RA by up-regulating the GAS5 and promoting the apoptosis of RA FLSs.

### Astragalosides

Astragalosides is a traditional Chinese medicine that has been proven to treat RA (Qi et al., 2017). After astragalosides treatment, the expression of 75 lncRNAs and 247 mRNAs changed, and the activity of 17 signal pathways changed. RT-qPCR and microarray data analysis showed that four lncRNAs (MRAK012530, MRAK132628, MRAK003448 and XR_006457) were key lncRNAs, which may be regulators under the action of astragalosides and the key therapeutic targets (Jiang et al., 2019).

### Caulophyllum Robustum Maxim

CRM was a traditional Chinese medicine for the treatment of RA in China (Wang et al., 2017a). A total of 218 significantly up-regulated genes and 191 down-regulated genes were identified between the CRME drug group and the control group, especially the Egr1, Cxcl2, Ccl3, Zfp36, Hist1h2ba and Hist1h2bj were involved in CRME’s regulation of RA pathogenesis. CRME mediate the course of RA through TNF signaling pathway, Toll receptor-like signaling pathway and chemokine signaling pathway (Lü et al., 2020).

### Quercetin

Quercetin is a dietary antioxidant and participates in the pathogenesis of cancer (Haleagrahara et al., 2017; Lu et al., 2020). Furthermore, quercetin up-regulated the level of lncRNA MALAT1. Knockdown of MALAT1 in RA FLSs reduced the expression of caspase-3 and caspase-9 and activated the PI3K/AKT signaling pathway, resulting in enhanced FLS proliferation and inhibition of FLS apoptosis. Therefore, quercetin promoted the FLS apoptosis by up-regulating the MALAT1 and inhibit the PI3K/AKT signaling (Pan et al., 2016).

### Tripterygium

Tripterygium is a traditional Chinese medicine used to treat RA (Zhang et al., 2017b). (5R)-5-hydroxytriptolide (LLDT-8) is a compound extracted from tripterygium wilfordii, which has lower drug toxicity and higher therapeutic effect (Zeng et al., 2016). Evidence suggested that LLDT-8 affected the gene expression network in FLSs of RA patients, especially the expression of lncRNAs and mRNAs in immune-related pathways. Comparing before and after LLDT-8 treatment, 394 genes were significantly differentially expressed in FLSs, of which 281 were down-regulated and 113 were up-regulated. Immune-related chemokine signaling pathway and TNF signaling pathway were involved in this mechanism (Guo et al., 2019).

### Huayu Qiangshen Tongbi Formula

HQT is a traditional Chinese medicine and is a commonly used Chinese medicine prescription for the treatment of RA (Wang et al., 2019c). The level of lncRNA uc.477 in the serum of RA patients was up-regulated, the level of miR-19b was down-regulated, and the former might have direct regulatory effect on the latter. Interestingly, HQT treatment of collagen-induced RA model mice normalized the expression of lncRNA uc.477 and miR-19b in FLSs of model mice. The therapeutic effects of HQT on RA might be achieved through the regulation of lncRNA uc.477 (Wang et al., 2020d).

## Conclusion and New Perspectives

LncRNAs are of great importance in gene regulation, almost participate in various biological process and pathways, and are closely related to the pathological mechanism of different diseases. It has become a research hotspot in the past few years and in the future (Dangelmaier and Lal, 2020). For the human genome, the number of lncRNAs produced is much greater than the number of coding RNAs (Chu et al., 2020). At present, except for the functions of a few lncRNAs in the pathogenesis of RA have been clarified, the effect and mechanism of most lncRNAs on RA are still unknown (Karami et al., 2020). It is worthy of in-depth study, which is of great significance for elucidating the pathogenesis of RA.

The research of lncRNAs in RA is an emerging field. At present, RA is the most studied in this field in autoimmune diseases, and less in other autoimmune diseases, such as systemic lupus erythematosus (SLE), Sjǒgren syndrome, scleroderma and polyarteritis nodosa (Long et al., 2016; Zhao et al., 2018; Cecchettini et al., 2019). In view of the common immune disorder and abnormal inflammation of these diseases, the role and mechanism of lncRNAs in RA have reference significance for the pathogenesis of other autoimmune diseases. However, a great number of studies have also found lncRNAs in various tissues are more tissue-specific than coding RNAs and microRNAs, indicating that lncRNAs are closely related to the functional specificity of tissues, which is a difficult point in the study of lncRNAs (Kazimierczyk et al., 2020).

Through high-throughput screening, the expression profile of lncRNAs in RA has been relatively elucidated (Sigdel et al., 2015). In-depth exploration of the function of lncRNAs in RA is extremely important. In addition to constructing lncRNA overexpression vectors or silencing lncRNAs to show the biological function of lncRNAs, it is also necessary to analyze how lncRNAs function in molecular mechanism, including the interaction between lncRNAs and RNA, the interaction between lncRNAs and proteins, and the binding of lncRNAs to DNA sequences (Akkipeddi et al., 2020). It is particularly important that in the initial research stage of lncRNAs, it is generally not considered that they have the ability to encode, but new evidence shows that some lncRNAs may function by encoding small peptides (Hartford and Lal, 2020). The coding potential of lncRNAs and the identification of peptides are currently hotspots.

The identified lncRNAs related to the pathogenesis of RA may become RA diagnostic markers or drug molecules that regulate disease progression (Wang et al., 2020c). However, it is still too early to develop disease-modifying therapeutics that directly target and regulate the lncRNAs, or whether lncRNAs themselves can be used as pharmaceutical molecules (Hu et al., 2020a). The technology and feasibility in this area are also immature for the earlier researched miRNAs (Tribolet et al., 2020). Moreover, siRNA-mediated treatment technology based on RNA interference has made progress. For example, the progression of animal model has been obtained by targeted knockout of related genes (Pearson and Jones, 2016). This provides inspiration for the development of lncRNAs as new targets for RNA interference-based therapies.

## Data Availability

The original contributions presented in the study are included in the article/Supplementary Material, further inquiries can be directed to the corresponding author.

## Glossary

ACPA: anticitrullinated protein antibody
CFA: complete Freund’s adjuvant
CRM: Caulophyllum robustum Maxim
CRP: C-reactive protein
DDR-2: discoidin domain receptor 2
DNMT1: DNA methyltransferase 1
ESR: erythrocyte sedimentation rate
EZH2: enhancer of zeste homolog 2
FER1L4: Fer-1-like protein 4
FLSs: fibroblast-like synoviocytes
FOXM1: forkhead box M1
GAS5: growth arrest specific transcript 5
HIPK2: homeodomain-interacting protein kinase 2
hnRNP Q: heterogeneous nuclear ribonucleoprotein Q
HQT: Huayu Qiangshen Tongbi formula
lncRNAs: long noncoding RNAs
PRC: polycomb repressor complexes
PBMCs: peripheral blood mononuclear cells
LLDT-8: (5R)-5-hydroxytriptolide
lnc-IL7R: Long noncoding-interleukin-7 receptor
LPS: lipopolysaccharide
MEG3: maternally expressed gene 3
NLRC5: nucleotide oligomerization domain (NOD)-like receptors 5: nucleotide oligomerization domain-like receptors 5
NLRC5: nucleotide oligomerization domain (NOD)-like receptors 5: nucleotide oligomerization domain-like receptors 5
PI3K: phosphoinositide 3-kinase
PVT1: plasmacytoma variant translocation 1
RA: rheumatoid arthritis
SCUBE2: signal peptide-CUB-EGF-like containing protein 2
sirt6: sirtuin 6
SLE: systemic lupus erythematosus
SMPD1: sphingomyelin phosphodiesterase 1
SNP: single nucleotide polymorphism
3′UTR: 3′untranslated region
VEGF: vascular endothelial growth factor A
